# Supplementary material for: Patterns of Intron Gain and Loss in Fungi
Source: PLoS Biol. 2004 Nov 30;2(12):e422. doi: 10.1371/journal.pbio.0020422 (PMC532390; doi:10.1371/journal.pbio.0020422)
Supplement: Table S1 — Also available at http://genes.mit.edu/NielsenEtAl/. (4.3 MB ZIP). [file pbio.0020422.st001.zip › NielsenEtAl/html/1390.html]

AN7027.1.NCU09027.1.MG02315.1.FG00787.1


```
 CLUSTAL W (1.82) Multiple Sequence Alignments - Introns Inserted


Sequence 1: NCU09027.1	487 aa
Sequence 2: MG02315.1	594 aa
Sequence 3: AN7027.1	455 aa
Sequence 4: FG00787.1	491 aa
Alignment Length: 597 aa
Number Identitical Residues: 224 aa
Alignment Score (without introns) 11413


MG02315.1 	MATPTEKE---NSVSGADKLDDVKLEVQ~PGQ~N~T0DEAPLTTFTAEEERALVWKQDMA
NCU09027.1	MAHSIEEM---DPDK-EKQLDEVEVSRN~-NE0F~C~QLTPEFEYSQAEEQALLRKQDLT
FG00787.1 	----MEKDPIPVPFPPPAAVDDVDSTLG~-KE~D0G~VAVHYGEAMSPLEKGLLWKQDMR
AN7027.1  	----MEKE---VAPDGVVNATDAELVIQ0---~-~-~-NSGGDAAVQAREKALIWKQDLH
          	     *:     .        :..                         *:.*: ***: 

MG02315.1 	ILPLSAAVYFLCYLDRSNIG~NAKILNSSTKNDMQSETHTTSYQFT~IALLVFLVAYAIF
NCU09027.1	ILPLCAAIYFLCYLDRSNIG~NARILNSSTHNDMQTETHSTQYQFN~IALMVFLVAYATF
FG00787.1 	IIPLSAAIYFLSFLDRTNIG~NAMILNSSTNNDMQRETNMSDHQFV~ISLMVFLVSYAVF
AN7027.1  	IIPLSAAIYLLCYLDRSNIG1NAKIMNSETGNDLLTETNMSSYEYT2IALMVFLIAYALF
          	*:**.**:*:*.:***:*** ** *:**.* **:  **: :.:::  *:*:***::** *

MG02315.1 	EVPSNILLKKLRPS~RWIAFLMFSWGAITIGLGGTSTFPQITGVRFLLGM~FEAGLFPG~
NCU09027.1	EVPSNILLKKLRPS~RWIAFLMFSWGAITMGLGGVQNFSSLAAVRFILGA~SEAGLFPG2
FG00787.1 	EVPSNMLLKKLRPS~RWLAFLMFCWGAMTILTAATNSFGSVTAVRFLLGV~FEAGLFPG~
AN7027.1  	EVPSNYFLKKLKPS0RWIAFLMLSWGAVTMGLGGAHNYAQVTGLRFLLGV1LEAGLFPG~
          	***** :****:** **:****:.***:*:  ... .: .::.:**:**   ******* 

MG02315.1 	LVYYLTFWYKAEERSVR~VAFILASATLAGA~FGGALAYAIGHMNGVHGLSAWRWLFILE
NCU09027.1	LVYYLTFWYKSDERSVR~VASILASATLAGA~FGGAIAYGIGHMNGASGLSAWRWLFIIE
FG00787.1 	LVFYLTFWYKHDERSVR2VAFILASATLAGA0FGGAIAYGIGHMNGVSGLSGWRWLFIIE
AN7027.1  	LVYFLTFWYRTSERSMR~VALILASATLAGA~FGGAIAYGVGHMNQAQGLSAWRWLFIIE
          	**::*****: .***:* ** ********** ****:**.:**** . ***.******:*

MG02315.1 	GLPSCLSAFAVWFLLPDFPEEWSRLSTRERDISHARLAIDGSKQHHSSMSWAEAKATLTD
NCU09027.1	GAPSCVSSLFVLFFLPDYPEEH--LKGREREIALARLKVEGSKSHHRSMTWQDAKTTLTD
FG00787.1 	GIPSCLAAFLVLFFLPDYPERAKWLSEAEKDMAIHRLYYEGSKESHPTMNWAEAKETLMD
AN7027.1  	GAPSLASAFLVWFILPDYPESASWLSEEEKALAAQRLAVEGSKGGAKAMTWQDAKEILLD
          	* **  ::: * *:***:**  . *.  *: ::  **  :***    :*.* :**  * *

MG02315.1 	WRLYGHYA~IYFGISTPFSSLSLFTPTITAGLGYFDLQAQLMTVPPYAVAYV1VQIITSY
NCU09027.1	WRLYGHYL~IYFGISVPFSSLSLFTPSITAGLGYKDLRAQLMSVPPYAVAYV1TQIVGSW
FG00787.1 	WRLYAHYA~IYFASGPAFASLSLFAPSITVGLGYFDLKAQLMTVPPWAIAYV1CQVLVAW
AN7027.1  	WRLYAHYV0VYFGISAPFSSLSLFTPAITSGLGYTSLNAQLMTVPPWAVAYV~VTTAVAW
          	****.**  :**. . .*:*****:*:** **** .*.****:***:*:***      ::

MG02315.1 	SADHFNARGIHSAVCATMGGIGFLVSGILPPSAFA~ARYGCLILGSAGAFSCIPPLLGWL
NCU09027.1	SADHFNARGIHSAVFSLLGAVGFIAMVTLPVDAYQ~HRYGCLILASAGSFACVPPMLGWL
FG00787.1 	SADHFNARGIHTAMAATVGAVGFIVSAALPADSYK~ARYGGLIMATAGSFACMPPMLGWL
AN7027.1  	SADHFNR----------------------YPRISL0HRYGCLIVATSGSFACIPPLLGWL
          	******                               *** **:.::*:*:*:**:****

MG02315.1 	SSNIFSTGAVGLAIAINVSIGGGLGQIPGVWIYKPEEAKLGYPTGHFTNAALLFFVAAGA
NCU09027.1	SSNVYSTASVGLAIAINVSVGGGIGQIPGVWIYPKEEAARGYPTGHWTNAGMLLMVSAGA
FG00787.1 	SSNVFTTASTGLAIALNVSIGGGIGQIPGVWIYQTSERQGGFTTGHWVNAALLLFVAVMA
AN7027.1  	SSNIRSTAGTGLAIALNVSFG-APGQIVGVWIYKSNEAKRGYPTGHWTNAALLLLVTVGC
          	***: :*...*****:***.* . *** *****  .*   *:.***:.**.:*::*:. .

MG02315.1 	LGLRFFYGWKNAKLLRENPGGN0ALLSITPFFTLALPPCVTQTPPATLQNNPPRLFRTTS
NCU09027.1	VGLRVWYGYKNRQLLRESNG--~---------------------------QEVRLFRL--
FG00787.1 	LGLRLFYGWKNRQLREYAEAHG~---------------------------VPYRCYKL--
AN7027.1  	LALRVYYGWRNKRLKAGGSG--~------------------------------QRFAY--
          	:.**.:**::* :*     .                                 : :    

MG02315.1 	LHHQWPCTPSTRPEPVMRQKQLVHPGISTSNTPRFVRRTPTFWAQPARSTVIHGGSYPDR
NCU09027.1	------------------------------------------------------------
FG00787.1 	------------------------------------------------------------
AN7027.1  	------------------------------------------------------------
          	                                                            

MG02315.1 	KSWSRLAQSRRR
NCU09027.1	------------
FG00787.1 	------------
AN7027.1  	------------
          	
```
